# 2368. SARS-CoV-2 mRNA vaccination induces B cell immunity in the tonsils and adenoids of children

**DOI:** 10.1093/ofid/ofad500.1989

**Published:** 2023-11-27

**Authors:** Qin Xu, Pamela Mudd, Hengameh Behzadpour, Lorenza Bellusci, Gabrielle Grubbs, Sara Pourhashemi, Juanjie Tang, Can Liu, Daniel Newman, Lihong Shi, Pedro Milanez-Almeida, Lela Kardava, John Tsang, Susan Moir, Surender Khurana, Pamela Schwartzberg, Kalpana Manthiram

**Affiliations:** National Institute of Allergy and Infectious Diseases, Bethesda, Maryland; Children's National Hospital, Washington, District of Columbia; Children's National Hospital, Washington, District of Columbia; Food and Drug Administration, Silver Spring, Maryland; Food and Drug Administration, Silver Spring, Maryland; Food and Drug Administration, Silver Spring, Maryland; Food and Drug Administration, Silver Spring, Maryland; National Institute of Allergy and Infectious Diseases, Bethesda, Maryland; Children's National Hospital, Washington, District of Columbia; National Institute of Allergy and Infectious Diseases, Bethesda, Maryland; National Institute of Allergy and Infectious Diseases, Bethesda, Maryland; National Institute of Allergy and Infectious Diseases, Bethesda, Maryland; Yale School of Medicine, New Haven, Connecticut; National Institute of Allergy and Infectious Diseases, Bethesda, Maryland; Food and Drug Administration, Silver Spring, Maryland; National Institute of Allergy and Infectious Diseases, Bethesda, Maryland; National Institute of Allergy and Infectious Diseases, Bethesda, Maryland

## Abstract

**Background:**

We recently reported that SARS-CoV-2 infection triggered robust adaptive immune responses in the tonsils and adenoids of children. However, whether intramuscular mRNA vaccination induces immunity in these tissues is unknown.

**Methods:**

We collected peripheral blood, tonsils, and adenoids from 21 children undergoing tonsillectomy/adenoidectomy in 2022 who had previously received a COVID-19 mRNA vaccination. From questionnaires and serology, we identified 10 vaccinated subjects who had not been infected; the remaining 11 were both infected and vaccinated. We characterized SARS-CoV-2-specific B cells that bound fluorescently-labelled spike proteins (S1 and RBD) in the blood and tissues of these participants using high dimensional flow cytometry, and compared them to SARS-CoV-2-specific B cells from 24 COVID-19 convalescent children recruited in 2020-2021.

**Results:**

We identified SARS-CoV-2-specific B cells (S1^+^RBD^+^) in the blood, tonsils, and adenoids of nearly all infected and vaccinated subjects. Tonsils and adenoids post-infection had a higher percentage of S1^+^RBD^+^ memory B cells than post-vaccination, but differences in the percentage of S1^+^RBD^+^ cells were not noted in the peripheral blood. Furthermore, we found that only 20% of vaccinated only subjects had SARS-CoV-2-specific germinal center B cells in their tissues compared to over 70% of infected subjects and subjects with hybrid immunity. Unsupervised analyses of the high dimensional flow cytometry data revealed differences in the characteristics of SARS-CoV-2-specific B cells post-vaccination and post-infection; S1^+^RBD^+^ B cells from infected subjects had a higher portion of CXCR3^+^ and IgA^+^ memory B cells, while those from vaccinated subjects had a greater proportion of CD21^lo^ memory B cells in the blood and tissues, implying a greater extrafollicular response post-vaccination but stronger mucosal IgA and IFN-γ-induced B cell responses post-infection.

**Conclusion:**

We found tissue-specific immunity to SARS-CoV-2 in the upper respiratory tract lymphoid tissue after mRNA vaccination, but vaccination induced B cell phenotypes distinct from that of natural infection which may affect the quality and duration of immunity in the blood and at the mucosal surface.

**Disclosures:**

**Pedro Milanez-Almeida, PhD**, Novartis: Salary

